# Status and Challenges of Plant-Anticancer Compounds in Cancer Treatment

**DOI:** 10.3390/ph14020157

**Published:** 2021-02-14

**Authors:** Paula Garcia-Oliveira, Paz Otero, Antia Gonzalez Pereira, Franklin Chamorro, Maria Carpena, Javier Echave, Maria Fraga-Corral, Jesus Simal-Gandara, Miguel Angel Prieto

**Affiliations:** 1 Nutrition and Bromatology Group, Analytical and Food Chemistry Department, Faculty of Food Science and Technology, University of Vigo, Ourense Campus, E-32004 Ourense, Spain; paula.garcia.oliveira@uvigo.es (P.G.-O.); pazoterofuertes@gmail.com (P.O.); antia.gonzalez.pereira@uvigo.es (A.G.P.); chamorro1984@gmail.com (F.C.); maria.carpena.rodriguez@uvigo.es (M.C.); ecalja@outlook.es (J.E.); mfraga@uvigo.es (M.F.-C.); 2 Centro de Investigação de Montanha (CIMO), Campus de Santa Apolonia, Instituto Politécnico de Bragança, 5300-253 Bragança, Portugal

**Keywords:** natural compounds, traditional plants, anticancer, clinical/pre-clinical studies, challenges

## Abstract

Nowadays, cancer is one of the deadliest diseases in the world, which has been estimated to cause 9.9 million deaths in 2020. Conventional treatments for cancer commonly involve mono-chemotherapy or a combination of radiotherapy and mono-chemotherapy. However, the negative side effects of these approaches have been extensively reported and have prompted the search of new therapeutic drugs. In this context, scientific community started to look for innovative sources of anticancer compounds in natural sources, including traditional plants. Currently, numerous studies have evaluated the anticancer properties of natural compounds derived from plants, both in vitro and in vivo. In pre-clinical stages, some promising compounds could be mentioned, such as the sulforaphane or different phenolic compounds. On the other hand, some phytochemicals obtained positive results in clinical stages and were further approved for cancer treatment, such as vinca alkaloids or the paclitaxel. Nevertheless, these compounds are not exempt of limitations, such as low solubility, restricted effect on their own, negative side-effects, etc. This review aims to compile the information about the current phytochemicals used for cancer treatment and also promising candidates, main action mechanisms and also reported limitations. In this sense, some strategies to face the limitations have been considered, such as nano-based formulations to improve solubility or chemical modification to reduce toxicity. In conclusion, although more research is still necessary to develop more efficient and safe phytochemical drugs, more of these compounds might be used in future cancer therapies.

## 1. Introduction 

**Figure 1:**
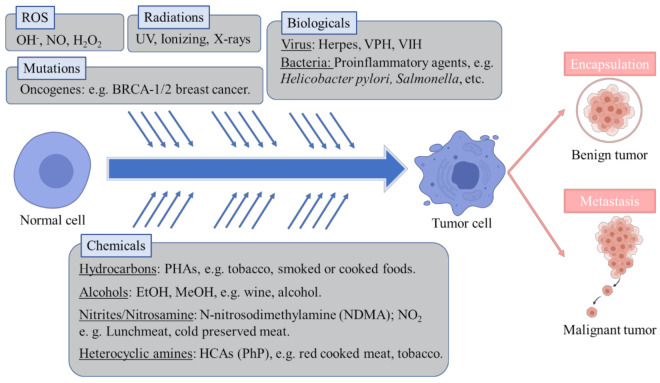
Main causes involved in the development of cancer, according to WHO.

Cancer is one of the deadliest diseases globally and especially in western countries. According to the International Cancer Observatory, roughly 9.9 million people have died in 2020 as a result of developing cancer [1]. Cancer is a complex disease, generally defined as an uncontrolled proliferation and development of cells in tissues forming an amalgamation and microenvironment (tumor) that may potentially expand to a whole organ or systemically to other tissues (metastasis) [2]. This abnormal cell behavior may be the result of hereditary genetics, or an epigenetic-driven alteration of key genes (oncogenes) related to the cell cycle and regulation of cell death (apoptosis) [3]. Cancerous cells are also characterized by dysregulation of programmed apoptosis and aberrant behavior of microtubules, as they are involved in the mitotic process [3]. The World Health Organization identifies as main causes behind the development of cancer random somatic mutations, ionizing radiation, reactive oxidative species as well as several chemical and biological agents [4]. Except for random mutations, these are widely recognized exogenous carcinogens. Ionizing radiation is able to disrupt the hydrogen bonds between nucleic acids as well as altering their chemical conformation, which may yield alterations in normal DNA expression regulation [5]. Infectious diseases caused by bacteria, fungi or viruses have also been significantly correlated with developing cancer afterward in the same affected tissues. Well-established associations between infections and cancer are viruses like human papillomavirus to cervix cancer, *Herpesvirus* to Kaposi’s sarcoma or Hepatitis B and C to liver cancer. In the same sense, bacterial infections by *Helycobacter pylori* are linked to gastric cancer or the genus *Salmonella* to colon or gallbladder cancer development post-infection [6]. Viruses that integrate their genetic material into the host may alter normal expression of genes related to cell division or even induce expression of oncogenes that could derive into oncogenesis. Some examples of these are the integration of Hepatitis B virus into telomeres, as well as genes coding for proteins X and S, which induce inflammation and neoplasia; or Human Papilomavirus integration in oncogenes E6 and E7 in the cell genome, which suppress the p53 anti-tumor gene and promote cell proliferation while simultaneously alters cell-to-cell adhesion [7,8,9]. These oncogenes have been related to increased release of inflammatory mediators like necrosis factor kappaB (Nf-κB) or Activator protein-1 (AP-1) [8]. Conversely, bacterial infections may elicit the release of toxins with cytotoxic activity and the disruption of the tissue cell matrix. Known examples are enteric toxins from *S. typhi* or CagA and vacuolating toxins of *H. pylori*, which may induce cell death, neoplasia, and also alterations in the normal cell metabolism [10,11]. Other infectious pathogens like fungi and parasitic helminths that produce direct or toxin-mediated tissular damage are also accounted as oncogenic agents [12]. Aside from specific genetic alterations, the main recognized tumor-inducing mechanism of biological agents is tissue inflammation as a result of cell damage and subsequent neoplasia which, if unchecked, can result in potential chronic inflammation of the affected tissues (e.g., hepatic cirrhosis by Hepatitis virus) [7,13]. Regarding reactive oxygen species, like hydroxide peroxide or hydroxyl radical, which are normal metabolic products but also arise from contact with oxygen, they are described to provoke damage and alterations of the cell membranes, lipids or DNA [14]. Indeed, reactive oxygen species have been identified to increase in tumorous cells, enhancing their proliferation and survivability [15]. Nonetheless, the common factor besides possible genetic alterations by oxidative stress, infections and ultraviolet radiation is the associated inflammatory response [12,14]. On this matter, chronic inflammation is considered both cause and symptom of other ailments, but particularly of cancer, as tumorous cells secrete several pro-inflammatory molecules [16]. For example, it is well known that the pro-inflammatory mediator cyclooxygenase-2 (COX-2) is overexpressed in several types of cancer. As such, pro-inflammatory mediators are markers of cancer and could be also a possible target for anticancer therapies [17,18]. Considering chemical carcinogens aside from potential hazardous substances, the main carcinogens originate in diet. Major chemical carcinogens include polycyclic aromatic hydrocarbons (PAHs), N-nitroso compounds, heterocyclic amines (HCAs) and alcohol. PAHs like anthracene appear in combustion reactions, and are reported in grilled or smoked foods, as well as being part of urban air pollution. They are linked to lung and digestive tract cancer [19,20]. Closely related in their effects and occurrence, HCAs like 2-Amino-1-methyl-6-phenylimidazo [4,5-b]pyridine are the result of pyrolysis of proteins and amino acids in meat or fish foods [21,22]. It is worth mentioning that tobacco is reported to contain high levels of PAHs and HCAs, linking them to the pro-carcinogen effects of tobacco consumption [23]. N-nitroso compounds are additives in processed meats and include nitrites and nitrosamines like N-nitrosedimethylamine that have been correlated to gastric cancer development [24]. Ethanol as well as other alcohols present in beverages and spirits induce many metabolic and endocrine disorders along with being highly cytotoxic chemicals and attributed to cause many types of cancers [25]. Altogether, it should be considered that a variety of exogenous carcinogens from different sources can heavily prompt cancer development (Figure 1).

Cancer not only displays heterogeneous cell and tissue-specific behavior, depending on the onset, inducer and individual genetic profile, but also an unpredictable and diverse evolution that hinders its treatment [26]. Conventional cancer treatment approach commonly involves chemotherapy, radiotherapy and surgery [27,28]. Regarding chemotherapeutics, their effect is cytostatic, acting through shifting the expression of cell cycle mediators, disruption of microtubules, or inducers of apoptosis. However, as radiotherapy and chemotherapy do not discriminate between normal and cancer cells, their application is paired with certain side effects, ranging from mild gastrointestinal alterations and nausea to severe gut mucosa dysfunction, cardiovascular toxicity or immunity disorders [29,30]. These side effects, which can linger for long periods after treatment, pose a major issue when selecting and applying therapeutics.

**Figure 2:**
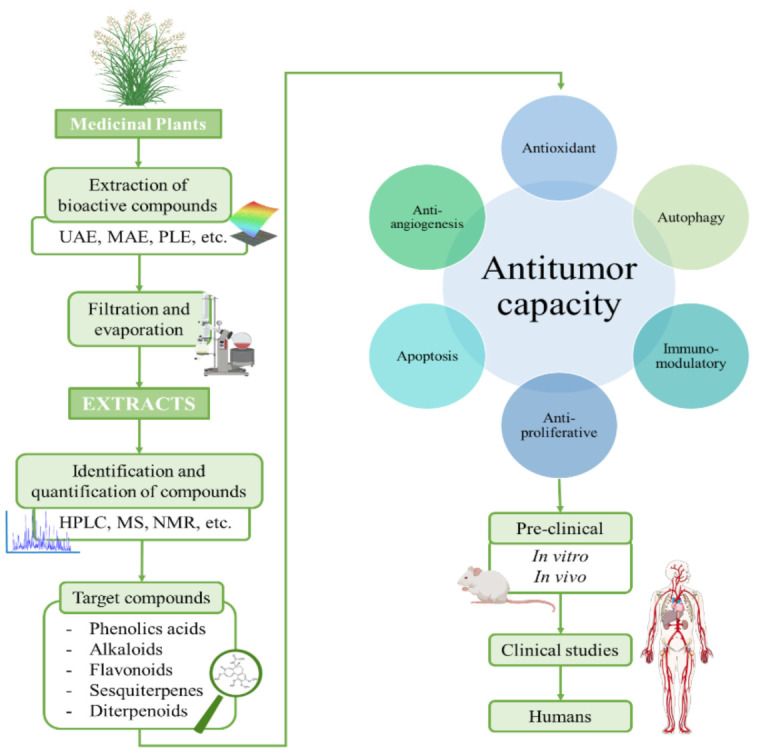
Schematic process for the development of anticancer drugs based on plant-derived compounds.

The ever-growing interest in the search of new therapeutic compounds against cancer has pushed researchers to look for innovative sources of anticancer compounds in natural sources, including plants [31]. Traditionally, plants have been used in all cultures for healing diverse diseases and improve well-being [32,33]. Further research demonstrated that traditional-used plants contain bioactive compounds, which administered in sufficient doses, have positive effects on health. These effects are attributed to the biological properties of the compounds, such as antioxidant, anti-inflammatory, antimicrobial and also anticancer. Nowadays, the potential of plants as sources of anticancer compounds is both well recorded in traditional medicine and experimental findings [34]. In several cases, phytochemical compounds have been directly employed or chemically modified to develop chemicals used in modern medicine, including anticancer drugs. According with the Food and Drug Administration (FDA), more than the 60% of the drugs employed in cancer treatment are obtained from natural resources [32]. In Figure 2, a schematic process for the development of anticancer drugs based on phytochemical compounds is presented. Briefly, this process starts with the extraction from plants and the testing of the extracts to evaluate their anticancer potential. Bioactive compounds of the extract are identified, purified and tested in pre-clinical studies, (both in vitro cell cultures and in vivo animal models) and later clinical trials in humans [35]. In these studies, some factors such efficacy, induced tumorigenic changes, possible side effects and toxicity factors must be deeply characterized [30]. Vinka alkaloids, taxanes, and camptothecin are some examples of compounds that are currently clinically employed.

In general, plant-anticancer compounds have been considered a possible option to develop new chemotherapeutics and also to enhance the effectivity of the conventional ones [36,37,38]. Nevertheless, these compounds present many drawbacks, such as low stability or solubility, difficulty to be extracted from natural sources and even negative side effects [39]. Thus, the application of these compounds still has to face several challenges and further research is necessary. In this review, current information about phytochemical compounds currently employed clinically in cancer treatment and also promising compounds in pre-clinical and clinical level will be addressed. In addition, the main challenges lying facing the use of these compounds as therapeutic agents and possible strategies to solve them will be described.

## 2. Phytochemicals Currently Used in Cancer Therapy

**Figure 3:**
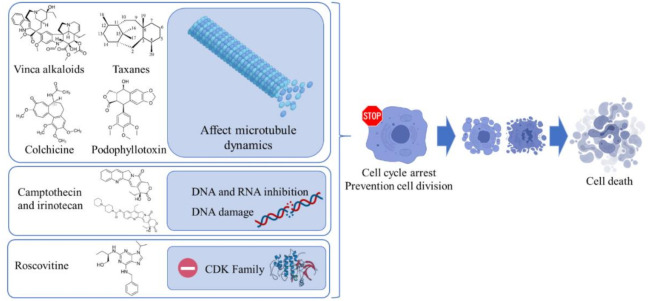
Main mechanisms of phytochemical compounds employed in cancer therapy.

Along the last decade, many works have compiled ethnomedicinal and ethnopharmacological uses of very different plant species. Numerous experimental works based on the evaluation through in vitro and in vivo assays have confirmed the therapeutic application of many natural compounds, which have been later included as part of approved treatments, including in anticancer agents (Figure 3) [40].

This section summarizes current available information regarding the clinical status of the main plant compounds proposed for cancer treatment. Vinka alkaloids, taxanes, camptothecin derivatives, podophyllotoxin and derivatives and roscovitine are the most used in clinical studies. Table 1 collects an overview of the data supporting the development of plant compounds as anticancer agents, including clinical trials and clinical uses.

### 2.1. Vinca Alkaloids

The vinca alkaloids is a large group of about 130 terpenoid indole alkaloids, naturally found in *Catharanthus roseus* leaves [31]. These compounds were one of the first plant alkaloids used in the development of anticancer drugs. Currently, several vinca alkaloids are in clinical use for cancer treatment: vinblastine, vincristine and also semi-synthetic derivatives, such as vinorelbine, vindesine, or vinflunine [45,46,47]. These compounds are considered small tubulin-binding molecules. The interaction with the tubulin proteins in the mitotic spindle avoids its polymerization into microtubules, inhibiting the cell mitosis. Thus, the cells remain in a prolonged arrest phase and finally die [48]. Vincristine has been demonstrated to possess highest affinity for tubulin than vinblastine, and even than the vinca derivatives vindesine and vinorelbine [47,49,50]. The dimeric nature of these alkaloids has been described to act through two ways. Vindoline moiety has been proposed to bind the β-subunit of tubulin heterodimers, which ultimately inhibits mitosis and leads to apoptosis. Catharanthine moiety has less potent effect on α/β-tubulin polymerization. However, different genes have been demonstrated to be sensitive to this molecule, such as those related with cell-to-cell signaling and interaction, which may be related with its capacity to induce cytotoxic effects [51].

In the 60s, Food and Drug Administration (FDA) approved the clinical application of vinblastine and vincristine, becoming very incipient plant-derived anticancer agents [40], usually combined with other compounds [52]. Vinblastine has been used in for the treatment of Hodgkin’s disease, apart from testicular carcinoma, breast cancer and germ cell tumors [53,54]. In a small phase II study of thalidomide and vinblastine in chemotherapy- refractory Hodgkin’s Lymphoma, both drugs demonstrated encouraging activity with some durable responses and acceptable toxicity [55]. In another phase II study carried out from 2001 to 2003 with patients posing advanced solid tumors, it was observed that the combination of daily cyclophosphamide and weekly vinblastine, administered concurrently with daily rofecoxib, provided moderate anticancer activity [56]. Moreover, the use of neoadjuvant chemotherapy with cisplatin, methotrexate, and vinblastine in patients with muscle-invasive urothelial bladder cancer treated by cystectomy and/or radiotherapy has been internationally investigated with 966 patients in the early 1990s [57]. The results showed that chemotherapy with cisplatin, methotrexate, and vinblastine improves outcome as first-line adjunctive treatment for invasive bladder cancer. Vinblastine was approved in Europe in 2009 for second-line treatment of metastatic and advanced urothelial cancer after failure of therapy with platin. Since then, the medicine was used in some clinical trials with successful results [58]. Vincristine has been used in chemotherapy in adults and children for treating acute leukemia but it has been also applied to treat rhabdomyosarcoma, neuroblastoma, Wilm’s tumor, Hodgkin’s disease and other lymphomas. Besides, it represents a treatment for non-malignant hematologic disorders like autoimmune and thrombotic thrombocytopenia, and hemolytic uremic syndrome.

### 2.2. Paclitaxel

In this group of molecules, the most recognized compound is paclitaxel, one of the most effective and promising therapeutic drugs available against a wide range of cancers. This compound has been identified in different species of the genus *Taxus* such as *Taxus brevifolia*, *T. baccata*, *T. media* or *T. cuspidate* [59]. It is an anti-neoplastic agent which acts as a microtubule’s stabilizer and mitosis inhibitor. Its main antitumor effect is derived from the compound binding the β-sub-unit of tubulin, which inhibits the microtubule depolymerization into tubulin and stops the cell cycle in the G2/M phase, leading to cell death [60].

FDA first approved Taxol^®^ for the treatment of ovarian cancer in 1992. Nowadays, the human prescription indications and uses of this drug comprise Kaposi’s Sarcoma, breast cancer, ovarian cancer, microcytic lung malignant neoplasm. In addition, it is often used to treat several cancers including oesophageal, bladder, prostate, cervical, gastric, head, neck and testicular cancers [61]. Nevertheless, paclitaxel has few drawbacks, such as low hydrosolubility, present side-effect and patients can develop resistance to the drug. Among them, the development of multidrug resistance that has limited its therapeutic efficacy has been not totally explained, although few potential mechanisms have been suggested, like α-/β-tubulin mutations and/or alterations in the binding regions [54].

### 2.3. Camptothecin and Irinotecan

Camptothecin is a natural compound with potent antitumor activity, isolated in 1966 from the bark of Camptothecin acuminata. This molecule binds to the TOP1 cleavage complex, leading to an accumulation of DNA strand breaks during replication, causing apoptosis in the S phase of the cell cycle [62]. The use of camptothecin in clinical applications has shown some limitations derived from its poor solubility and stability [63]. For this reason, several stable derivatives are currently under study in clinical trials, from which only two can be used in clinical studies by the US Federal Drug Administration. One of them is irinotecan, approved in 1996 as a therapy agent for refractory colorectal carcinoma [64]. This compound is now widely used to treat other types of cancer, including lung, although the dose limiting toxicity of this antitumor agent includes diarrhea, neutropenia and severe leukopenia [65]. The second compound is Topotecan, approved in 2007 as a secondary agent for therapy of ovarian cancer or small cell lung cancer [66]. However, some studies have revealed that therapeutic use of irinotecan is limited due to its hydrophobicity, low stability at physiologic pH, and side effects.

### 2.4. Podophyllotoxin and Analogues

Podophyllotoxin is an important plant-derived natural product isolated from *Podophyllum peltatum* and *Podophyllum emodi* [35]. It has a lignan as interesting molecule, which can be semisynthesized commercially, showing antitumor activity against different types of cancers, such as cervical carcinoma, osteosarcoma, nasopharyngeal carcinoma, colon cancer, breast cancer, prostate cancer, small cell lung cancer and testicular carcinoma [67]. The mechanism of action of podophyllotoxin is related to the blockage of cell division in the metaphase of mitosis due to the inhibition of microtubule assembly in the mitotic apparatus [35]. However, their derivatives etoposide and teniposide are not inhibitors of microtubule assembly, but act via interaction with DNA and inhibition of DNA topoisomerase II [35].

The initially clinical use of podophyllotoxin was declined rapidly, due to its unacceptable side effects, including nausea, vomiting, and damage to normal tissues [68]. However, due to its remarkable inhibitory effect on tumor cell growth and extensive use in traditional medicine, podophyllotoxin has constituted an important starting point in the development of three of the most highly prescribed anticancer agents worldwide: etoposide, teniposide, and the water-soluble prodrug etoposide phosphate [68]. The podophyllotoxin derivatives are gaining attention for clinically trials having wider use of podophyllotoxin scaffolds in medicine. In a randomized clinical trial, the effects of podophyllotoxin on anogenital warts with imiquimod 5% cream were compared. The study led to the conclusion of a strong inhibitory effect on warts growth in patients treated with podophyllotoxin versus patients treated with imiquimod cream [69].

### 2.5. Roscovitine

The agent Roscovitine (generic name Seliciclib) is a purine-based anticancer compound, isolated from the cotyledons of *Raphanus sativus* L. (Brassicaceae) [31]. This compound is a potent inhibitor of cyclin-dependent kinases (CDK) CDK1, CDK2, CDK5, CDK7, CDK9 and CDK12, through direct competition at the ATP-binding site [70,71]. As an inhibitor of the CDK family, roscovitine has an important impact upon several fundamental processes in cells [72]. CDK1 and 2 are involved in the control of the initiation and progression of cellular division. Then, the inhibition of these kinases leads to the reduction of the cell proliferation. The inhibition of CDK7, CDK9 and CDK12 results in downregulation of a large number of genes [71]. This compound has been tested in several phase I and II clinical trials showing its anticancer activity. Roscovitine was used in phase I clinical trial against glomerulonephritis, in phase II clinical testing against lung and breast cancer and currently, it is in a phase II clinical trial in patients with Cushing disease [73]. Roscovitine has also been tested in a phase I trial in combination with gemcitabine and cisplatin in non-small cell lung cancer patients and the results revealed that the 70% of the patients with nasopharyngeal tumors showed stable disease during the study [72]. Another example supporting the anticancer activity of this molecule is a phase I trial study in which roscovitine was orally administrated to patients with refractory solid tumors. In this case, although no tumor reductions were observed, roscovitine induced a stable disease in some patients [72]. Other studies in vivo revealed that roscovitine causes significant selective DNA damage in head and neck cancer cells and in human papilloma virus-positive (but not in human papilloma virus-negative) [74]. These results support the potential of roscovitine as a drug against human papilloma virus and head and neck squamous cell carcinoma. However, a phase II trial performed with almost 200 patients suffering with non-small cell lung cancer has failed. The study has met the primary endpoint of improving progression free survival, although patients administered with roscovitine did show longer median survival [72].

## 3. Compounds in Pre-Clinical and Clinical Stages

Numerous phytochemical compounds have demonstrated to exert significant anticancer activity in pre-clinical studies (both in vitro and in vivo). These positive results led to their evaluation in further clinical trials, to estimate the suitability (preliminary efficacy, toxicity, pharmacokinetics, safety data, etc.) of the compounds as possible agents in new strategies for cancer therapy (several examples have been compiled in Table 2). In this section, some of these compounds will be described, including colchicine derivatives, sulphoraphane and dietary phenolic compounds, such as resveratrol, curcumin, quercetin, gingerol and kaempferol. Nowadays, several pre-clinical and clinical studies have been conducted with these compounds, but more clinical trials are still necessary before their further application.

### 3.1. Colchicine Derivates

Colchicine is a natural alkaloid that was initially isolated from plants that belong to the genus *Colchicum* such as *Colchicum autumnale*. Later, its presence was also confirmed in other vegetal species like those from genera *Gloriosa* and *Sandersonia*. This compound is considered a tubulin poison because it suppresses microtubule dynamics, which ultimately disrupts the formation of the mitotic spindle. Colchicine, through its specific colchicine domain-binding, gets bound to tubulin between both α and β subunits which subsequently stimulates the depolymerization of tubulin complexes. This destabilization of microtubules is capable of arresting cell cycle at different phases leading to a final induction of the cell apoptosis [90]. Colchicine was initially evaluated as cancer therapy, but it presents few drawbacks: it possesses high toxicity and shows low specificity for tumor cells that lead it to target normal cells. Hence, colchicine has a limited medical application in cancer therapy. Nevertheless, semisynthetic derivatives that present lower toxicity have been developed and successfully applied on in vitro studies. Colchicinamide, deacetylcolchicine or valyl colchicine and other synthetic derivatives have been tested in different in vitro human cancer cell lines such as colorectal, chronic granulocytic leukemia, melanoma, central nervous system and breast cancers [36,91,92,93]. It seems that deacetylcolchicine has been employed in clinical trials, due to its effectiveness against melanoma, Hodgkin’s lymphoma, and chronic granulocytic leukemia [94], but, to our knowledge, no other clinical trials with colchicine derivatives have been reported. Most of the studies evaluating colchicine derivatives have been carried out in vitro. Some of these compounds have been considered more active and selective, compared to colchicine [92,93], but more pre-clinical and clinical trials are still necessary to assess the efficacy, pharmacokinetics, limitations and safety issues of these compounds, before considering them possible candidates for future cancer treatment.

### 3.2. Sulforaphane

Sulforaphane (1-isothiocyanate-4-methyl-sulfinylbutane) (SFN) is the best-known compound of the isothiocyanate group [95]. It is a redox-active natural molecule isolated for the first time in 1958 from the leaves of hoary cress (*Lepidium draba*). Afterwards, in 1992, it was found in plants from the Brassicaceae family. Today, this compound is well known to be found in foods and specifically in cruciferous vegetables like broccoli sprouts [96]. In fact, some studies show that extracts from broccoli can induce the phase II detoxification enzymes and the antioxidant enzymes in mammalian cells [97]. SFN has been tested for cancer prevention, cancer therapy and also in other chronic disease prevention and treatment bioassays [98]. This compound has regulatory effects on the tumor cell cycle, apoptosis, and angiogenesis by modulation of the related signaling pathways and genes [99]. In the analysis of the cell cycle, it was observed that SFN causes G2/M phase arrest, leading to inhibition of tumor proliferation/growth, which is associated with downregulation of cyclin B1 and cyclin D1 genes (related with the progression of the cell cycle), as well as increased protein levels of cyclin/CDK inhibitor p21 (related with cellular senescence). SFN also increased the expression of the pro-apoptotic protein Bax and decreased expression of the anti-apoptotic protein Bcl-x, inducing apoptosis in cancer cells [99].

In the last years, the anticancer properties of this compound have been demonstrated in vitro and in vivo, and a few clinical trials have been carried out tenting the suitability of oral-supplementation of SFN for cancer chemoprevention and also in combination with other anticancer drugs [100,101]. Clinical trials have been performed on prostate and breast cancer patients. In a double-blinded, randomized, placebo-controlled trial, the oral administration of SFN lead to the significant reduction of prostate-specific antigen in prostate cancer patients after radical prostatectomy [75]. In a phase II clinical trial on prostate cancer patients with biochemical recurrence after prostatectomy, patients who received oral administration of SFN showed a lengthening of the on-treatment prostate-specific antigen doubling time, compared to pre-treatment [76]. In a recent phase II clinical trial, the results showed that the administration of SFN as adjuvant enhanced the efficacy of doxorubicin against in vivo breast cancer [77]. More studies about clinical uses of SFN in humans seems warranted, due to the high availability and tolerability of this compound, and its effectiveness as a chemoprevention agent in preclinical models of carcinogen-induced cancer [102].

### 3.3. Phenolic Compounds

Some of the most promising compounds that may be in the development of new drugs are phenolic compounds. These compounds are secondary metabolites from plants (an also some algae), which could be divided into phenolic acids, flavonoids, lignans, stilbenes and other compounds [103]. Nowadays, these compounds have attracted the attention of scientific community due to their large range of biological properties, such as antioxidant, anti-inflammatory, antibacterial and also anticancer [104,105,106,107]. Due to their beneficial effects on health, the interest of the consumption of phenolic compounds has increased [108]. Regarding anticancer activity, it has been described that these compounds may interfere with the initiation, growth and progression of cancer by modulating different enzymes and also diverse signaling pathways related with cell-proliferation [105]. Several examples of phenolic compounds employed in pre-clinical and clinical studies have been described below.

#### 3.3.1. Resveratrol

Resveratrol is a phenolic compound present in some fruits, such as grapes, peanuts, blueberries and blackberries. Numerous studies have evaluated the anticancer properties of this compound. Several action mechanisms of resveratrol have been described: positive regulation of p53 and BAX proteins (related with pro-apoptotic pathways) and negative regulation of NF-κB, AP-1, hypoxia-inducible factor 1-alpha (HIF-1α), matrix metalloproteases, Bcl-2 protein, COX-2, cytokines and CDK [109]. Some pre-clinical studies performed in vitro demonstrated that resveratrol was able to suppress the cell proliferation through cell cycle arrest, induce apoptosis and modulate autophagy in different cancer cell lines, including ovarian cancer cell line, resistant human leukemia cells, non-small-cell lung cancer and human lung adenocarcinoma [110,111,112]. Regarding in vivo studies, the anticancer properties of this compounds were also significant. For example, in an in vivo study, resveratrol was administered to mice, leading to a 60% reduction in the appearance of sporadic colorectal cancer. Similarly, resveratrol inhibited cell proliferation, induced the apoptosis and suppressed the angiogenesis and metastasis in bladder cancer mice-models [113]. Resveratrol has been also reported to enhance the efficacy of traditional chemotherapeutic drugs, including temozolomide, doxorubicin and paclitaxel in mice models [114,115,116].

The positive results obtained at pre-clinical level led to the development of clinical studies using this compound. For example, in 2011, a phase I study conducted with colorectal cancer patients with hepatic metastasis reported that the administration of microparticular-formulated resveratrol (5 g/day, 14 days) increased the apoptosis of malignant cells in the hepatic tissue, compared with placebo [78]. In colorectal adenocarcinoma, resveratrol reduced the expression of COX-2 and M_1_G (NCT00433576). In another study, the results showed that trans-resveratrol (100 mg/day, 12 weeks) reduced the DNA methylation of RASSF-1α, a gene related with breast-cancer, and also reduced the cancer promoting prostaglandin E2 expression in the breast [79]. A phase I study conducted with recurrent prostate cancer patients with elevated prostate-specific antigen levels evaluated the effects of the administration of pulverized muscadine grape skin extract containing resveratrol (4000 mg). The results showed a significant reduction of the recurrence of the cancer by more than 5 months, compared with placebo [80]. A further 12-month study reported similar results using two different doses, 500 and 4000 mg of resveratrol [81]. Regarding these clinical trials, the use of resveratrol may be promising in cancer treatment. However, one of the main drawbacks of resveratrol is its low water-solubility, which limits its clinical use. To overcome this limitation, some strategies have been considered such as synthesis of novel derivatives and analogues [116], micro-formulation [78] or nano-delivery systems [117].

#### 3.3.2. Curcumin

Curcumin is a natural pigment of phenolic nature extracted from *Curcuma longa.* This compound has been extensively studied due to their immunomodulatory and anticancer properties, among others [118]. Numerous biological targets of curcumin have been described, such as proteins involved in the antioxidant-response, apoptosis, cell-cycle regulation and cancer progression [119]. Numerous pre-clinical studies have demonstrated that curcumin is an effective chemopreventive agent and also acts as a beneficial adjuvant [120]. To cite some examples, in vitro assays reported that curcumin exerted anticancer effects (reduction of cell proliferation, cell cycle arrest, apoptosis, autophagy, etc.) in different cells lines, such as human endometrial, malignant glioma, malignant mesothelioma, breast adenocarcinoma, melanoma, chronic myelogenous leukemia or gastric cancer cell lines [116,121,122,123,124]. In vivo studies have reported similar results, for example, in chronic myeloid leukemia [123], pancreatic cancer [125] or breast cancer mice models [126]. Curcumin has been also reported to enhance the effects of conventional drugs, such as cisplatin, doxorubicin, paclitaxel or irinotecan [127,128]. Nevertheless, this compound present low bio-availability, due to its low solubility, which limits its clinic applications. In this sense, nanoparticles-based drugs or derivative compounds have been reported to be suitable options to developed curcumin treatments [119,126].

Despite bioavailability limitations, to date, several clinical studies have evaluated the potential of curcumin against different cancers: breast, prostate, pancreatic, colorectal and hematological [35,116]. More recently, a study assessed the efficacy of curcumin complexed with phospholipids as complementary treatment of gemcitabine on pancreatic cancer patients. The results showed that the administration of the product (2000 mg/day) increased the efficacy of gemcitabine, without any observed toxicity [82]. Similar effects were observed in a II phase study, where curcumin was tested as complementary treatment of paclitaxel in metastatic and advanced breast cancer. The treatment with both compounds was more efficient compared with paclitaxel-placebo and no toxic effects were observed [83]. As a function of these results, it seems guaranteed that curcumin could be used in combination with the conventional drugs to enhance their efficacy in the future.

#### 3.3.3. Quercetin

Quercetin is a flavonoid commonly found in berries, onions and leafy vegetables, to which anticancer properties are attributed [129]. In this sense, numerous studies in vitro and in vivo pre-clinical studies have showed positive results. Regarding its action mechanisms, quercetin has been demonstrated to induce cell cycle arrest by regulating cyclin D1 and p53-related pathways; apoptosis trough the induction of pro-apoptotic factors and the decrease of anti-apoptotic ones; induces autophagy and inhibits proliferation, angiogenesis and metastasis [130,131]. These effects have been observed in different in vitro cell lines, including breast, ovarian, lung and colon cancer cells, among many others [130,132,133], and also in different in vivo mice models [134,135,136]. Furthermore, quercetin has been reported to enhance the efficacy of chemotherapeutic drugs [137,138].

Regarding clinical trials, several have evaluated the suitability of quercetin as anticancer drug. For example, a study conducted on humans reported that a high intake of quercetin in the diet is inversely related to the risk of gastric adenocarcinoma [84]. Another study evaluated the use of quercetin to prevent and treat oral mucositis induced by chemotherapy. The results showed a significant reduction of oral mucositis incidence in the quercetin treated group, which may suggest that this compound could be used to palliate chemotherapy side-effects [139]. Nowadays, some clinical trials are ongoing in prostate (NCT01912820), squamous cell carcinoma (NCT03476330) and lung cancer (NCT04267874). However, in some cases, the results are limited due to the low solubility and bioavailability of this compound. To overcome these drawbacks, several delivery systems have been proposed for instance, lipids, nanoparticles, nanoemulsions, liposomes or micelles [131].

#### 3.3.4. Gingerol

Gingerol is another phenolic compound with significant anticancer properties, extracted from the fresh rhizome of *Zingiber officinale*. The action mechanisms attributed to gingerol include reduction of inflammation (decrease of NFκB and COX-2), induction of phase II detoxification enzymes (Nrf2), genomic instability (tubulin interactions), and altered gene expression (increased expression of pro-apoptotic molecules and decreased expression of anti-apoptotic factors), among others [140]. These effects have been corroborated in different in vitro cultures, such as neuroblastoma, sarcoma, human myeloid leukemia, breast Caco-2 cancer cells, and also in breast cancer-animal models [141,142,143,144,145]. Recently, studies have demonstrated that gingerol may be used in combination with cisplatin [146] or doxorubicin [145], to improve their efficacy.

Several clinical trials have employed ginger root extracts, containing gingerol, to determinate its ability to prevent cancer development. In a pilot study, patients at increased risk for colorectal cancer received 2 g of ginger (standardized to 5% of gingerol). The results suggested that ginger supplementation might be effective to prevent the apparition of cancer [85]. In other pilot study, newly diagnosed cancer patients received a ginger extract (standardized 6-gingerol 20 mg/day) as chemotherapeutic adjuvant, starting 3 days prior to the first cycle of chemotherapy till the fourth cycle. The results obtained showed that the antioxidant status was increased and oxidative markers were reduced in the ginger group, suggesting that daily supplementation could reduce the negative effects of chemotherapy [86]. Recently, a phase II, randomized, double-blind, placebo-controlled study confirmed that 6-gingerol significantly improved the overall complete response rate in chemotherapy-induced nausea and vomiting, appetite, and quality of life in cancer patients receiving chemotherapy [87]. It has been described that gingerol has low solubility in water, leading to low bioavailability and limited applications. Several strategies may be employed to overcome these drawbacks, such as proliposomes [147], nanostructured lipid carriers [148] or microemulsion [149], achieving higher bioavailability and activity, compared to free gingerol.

#### 3.3.5. Kaempferol

Kaempferol is one of the most common flavonoids, found in diverse fruits and vegetables. Many studies have described the beneficial effects of dietary kaempferol in reducing the risk of chronic diseases, especially cancer [150]. Kaempferol increases the body’s antioxidant defense against free radicals, which have been demonstrated to be one of the main causes involved in cancer development. At molecular level, kaempferol has been reported to modulate several key elements in cellular signal transduction pathways linked to apoptosis, cell-cycle arrest, angiogenesis, inflammation and metastasis [150,151]. Nowadays, several pre-clinical in vitro and in vivo studies have reported the anticancer properties of this flavonoid against different types of cancers, including breast, prostate, ovarian, lung, colon or kidney [152].

Several clinical studies have evaluated the relation between the intake of dietary flavonoids (including kaempferol) and cancer, showing variable results. Many studies have reported no significant relation between oral intake of kaempferol and risk of cancer development [153], ovarian [154] or breast cancer [155]. On the other hand, some clinical studies have reported significant reduction in ovarian cancer [88] and the risk of pancreatic cancer development in smokers [89]. Considering these results, it would be necessary to perform more clinical studies with larger groups. In addition, like other phenolic compounds, the clinical applications of this compound are limited by its poor bioavailability. In this sense, nano-formulations, phospholipids or metallic complex could be strategies to enhance the bioavailability of these compounds and thus, their bioactivity [152,156].

### 3.4. Other Plant Derived Compounds

Other plant-derived compounds that have demonstrated anticancer properties both on in vitro and in vivo studies have been compiled in Table 3. The results obtained in pre-clinical stage could led to the development of more clinical studies and the design of new chemotherapeutics.

## 4. Current Challenges of Anticancer Phytochemicals

The development of phytochemical anticancer agents requires substantial evidence of efficacy derived from adequate clinical trials before their approval for use in patients. Although the mentioned compounds present significant anticancer properties, they also have limitations that should be overcome before its further clinical application, in the case of compounds in clinical stage, or to enhance their characteristics, in the case of drugs currently used in cancer treatment. In this sense, poor aqueous solubility, poor penetration to enter in targeted cells, absorption by normal cells, limited therapeutic potential and toxic side effects are still the major concern of the application of phytochemical compounds in cancer treatment [35,211]. For example, compounds like colchicine, camptothecin and podophyllotoxin derivatives have showed limited use due to the side effects. In some cases, compounds such as SFN or vinca alkaloids present limited effect and they are used in combination with other agents. In this section, several challenges and strategies proposed to solve the drawbacks of these compounds will be discussed. Apart from these challenges, the discovery and development of possible phytochemical compounds employed clinically also has to face other challenges related to the extraction synthesis, optimization and characterization of phytochemicals anticancer compounds. Thus, new advances in analytical technology and computational methodologies, to facilitate the identification of new phytochemicals, optimize their extraction and/or opt for its chemical synthesis or modifications are expected.

### 4.1. Limited Solubility and Poor Absortion

As mentioned in previous sections, some compounds present low availability, which is one of the main reasons of the limited potential of phytochemicals. To solve bioavailability limitations related to stability, absorption and metabolism, lipid-based formulations (such as microencapsulation, liposomes, nano-emulsion, etc.) and nanotechnology have been considered as a suitable approach to enhance aqueous solubility, stability, target specific to cancerous cells and even reduce the quantity of the phytochemical compound to achieve secure therapeutic levels [211,212]. For example, the administration of the vinca alkaloids has been further improved by including them into micro- or nano-capsules, such as liposomes or micelles (“Liposome-encapsulated vinca alkaloids and their use in combating tumors” patent no. US4952408A; “Nano anticancer micelles of vinca alkaloids entrapped in polyethylene glycolylated phospholipids” patent no. US8765181B2). The encapsulation of core ingredients permits a controlled release and protects the bioactivity from external factors that may affect the performance. Among them, liposomes named after Marqibo^®^, have been approved by FDA to treat adult leukemia [54]. Similar efforts have been conducted to improve the solubility of paclitaxel, and several nano-technological platforms have been developed including polymeric nanoparticles (Abraxane^®^), polymeric conjugates (Xyotax), polymeric micelles (Genexol-PM, NK 105) and liposomes (LEP-ETU). However, although a large number of nano-medicines has been investigated in the last decades, only few nano-formulations have achieved their use in clinical trials, with even fewer being approved for clinical application. For example, the hydroxypropyl-methacrylamide copolymer–PTX developed by the pharmaceutical Pfizer has stopped in Phase I due to the high neurotoxicity in rats during the study. Poly (L-glutamic acid)-paclitaxel (PG-TXL) belongs to the few formulations that reached phase III clinical trials. Unfortunately, the development of PG-TXL stopped in 2016 due to the inability to show significant improvement over current standard care [213]. In the case of irinotecan, some studies have revealed that its therapeutic use is limited due to its hydrophobicity, low stability at physiologic pH, and side effects. These issues could be solved by new drug formulations containing nanoparticles, polymer conjugates, dendrimers, peptides, and carbohydrates [64].

Regarding compounds in clinical studies, as previously mentioned, low solubility and availability are some of the main limitations that hinder their possible use for cancer treatment. For example, it has been reported that resveratrol has a circulation half-life of several minutes, while quercetin is generally present at low micromolar concentrations in blood, insufficient for a potent anticancer activity [214]. Thus, numerous studies have evaluated innovative strategies to enhance their availability, including nano-formulations, liposomes, nanoparticles, etc. For example, gingerol formulated in proliposomes showed a higher inhibition of HepG2 cancer cells in vitro and also a higher oral bioavailability in vivo [147]. In other study, gold-coated iron oxide nanoparticles improved significantly the stability and activity of SFN against human breast cancer cells [215]. Recently, mesoporous silica nanoparticles conjugated with hyaluronic acid were employed as a drug delivery system of curcumin, and the anticancer activity was measured in vitro and in vivo. The results showed that the delivery system enhanced the anticancer activity in both assays, which may attributed to a higher bioavailability [216].

Nowadays, several clinical trials have been performed using innovative strategies to enhance the bioavailability of chemotherapeutic drugs, as recorded in other reviews [212,214]. However, these studies are few compared with the numerous works published evaluating novel strategies. Therefore, it is expected that more clinical trials will be conducted in the future.

### 4.2. Combination of Phytochemicals with Conventional Chemotherapeutics

Some authors have pointed out that cancer treatment with a single-target chemical agent is not entirely effective since it is difficult to achieve optimal cytotoxic effects in cancer cells due to their rapid molecular adaptations. In addition, some phytochemicals currently in clinical evaluation present limited effects on their own [217]. Therefore, a combinatorial approach of these compounds with conventional chemotherapeutics may achieve a synergistic effect that increases toxicity in cancer cells (associated to the sensitization of the cancer cells to the drugs, attributed to the phytochemicals), reduces therapeutic doses and also toxicity concerns [214,217,218]. In this sense, numerous studies have evaluated the efficiency of this combination and have been compiled extensively in recent reviews [116,219]. For example, resveratrol has been successfully used as adjuvant of several conventional chemotherapeutic drugs, such as temozolomide, doxorubicin and paclitaxel on in vitro assays and mice models [114,115,116]. Other compound commonly employed as chemosensitizing agent is curcumin. For example, when combining curcumin with docetaxel, a decrease in drug resistance was observed in breast cancer cells [220]. Similar results were also observed combined with vincristine, paclitaxel, irinotecan and cisplatin, enhancing the efficacy of these drugs, compared to single treatment [127,128,221,222]. Recently, gingerol has been found to enhance the sensitivity of in vitro gastric cancer cells to cisplatin [146], and the sensitivity of liver cancer cells to doxorubicin [145]. Other compounds that have been employed in combinatory assays appeared in Table 3, like apigenin, betulinic acid and berberine. In addition, as could be observed in Table 2, the combination strategy has been also assessed in clinical trials. Thus, although more studies are still necessary, it seems that the use of phytochemicals as chemosensitizing may be beneficial in cancer therapy.

## 5. Conclusions

Traditional plants have been historically considered as an endless source of new compounds for the development of new pharmaceuticals and drugs. Therefore, nowadays researchers have at their complete disposal, plenty of ethnomedicinal and ethnopharmacological information of very different plant species which is a tool for selecting candidates and lead the research to those plants more promising. As a result of this knowledge, different examples of phytochemicals with medicinal properties against different diseases can be found throughout the literature.

Cancer is a complex disease that every year costs several millions of human lives. The uncontrolled proliferation of cells causes the incorrect functioning of the body, with a long list of symptoms and finally, death. So, given the health and social important of this disease, but also its economic impact on the health system, new therapeutic alternatives are being continuously investigated. For this purpose, it is essential to consider some factors such as clinical trials, efficacy, the induced tumorigenic changes, possible side effects and toxicity of the pharmaceuticals. In this context, a variety of phytochemicals obtained from plants have been discovered and are currently used in cancer therapies. In the present review, some examples such as vinca alkaloids, paclitaxel or irinotecan, that have demonstrated to exert anticancer effects on clinical trials and have been approved for clinical use are collected. Nevertheless, these compounds are not exempt of limitations, such as low solubility, restricted effect on their own, negative side-effects, etc. This article also highlights some compounds that are being investigated in pre-clinical and clinical stages, such as SFN and different phenolic compounds, which have shown interesting results. However, more clinical trials are still necessary before their further safe application.

At last, after a brief discussion of the information collected on the manuscript, an attempt has been made to identify which are the main challenges of anticancer phytochemicals, current needs and future research lines. Low availability is a target challenge for this area, which is now focused on the development and improvement of drug delivery systems, such as nano-based formulations. Moreover, many of these compounds have been demonstrated to be effective chemosensitizing agents, so they could be used combined with conventional chemotherapeutics. In this sense, more clinical trials evaluating the efficacy of the systems to enhance availability and combinatory trials should be performed before considering a compound for its use alone or in combination with other drugs.

The manuscript brings to light the need for more large-scale clinical trials and standardization procedures to validate phytochemicals in anticancer therapy. Thus, it is essential the coordinate work of interdisciplinary areas like medicinal chemistry, pharmacology, biochemistry and biology to deeply understand the availability, efficacy, safety, mechanism of action and synergistic effects of these new drugs. Therefore, to address all the limitations and needs of this research area, this review aimed at compiling the information about the current phytochemicals used for cancer treatment, promising candidates and to identify those fundamental challenges for the development of new alternatives.

## Figures and Tables

**Table 1:** Anticancer compounds employed in clinical treatment of cancer [41,42,43,44].

Compounds	Source-Extraction	Mechanism Action	Clinical Development	Commercial Name
Vinca alkaloids	*Catharanthus roseu* (Leaves) Isolated by semi-synthetic routes	Inhibit the tubulin polymerization of tumor cells and also cause mitotic spindle destruction	In clinical use; combination trials	Vinorelbine, Vincristine, Vinblastine, Vindesine, Vinflunine, Vincamine, Vintafolide
Paclitaxel, docetaxel	*Taxus* spp. (Bark) Synthesis, semi-synthesis, and plant cell culture	Stabilization of microtubules and inhibition of depolymerization into tubulin, which stops the cell cycle in the G2/M phase leading to cell death	In clinical use; Phase I-III clinical trials; early treatment settings; non-small lung cancer, breast cancer, ovarian cancer, Kaposi sarcoma. Research and development in alternative drug administration using nanoparticles, naocochealtes and nanoliposomes.	Taxol^®^, Taxotere^®^, Abraxane^®^, Jevtana^®^, Taxoprexin^®^, Xytotax^®^
Camptotecin, irinotecan	*Camptotheca acuminata* (leaves) Water extraction	Binding to the TOP1 cleavage complex, leading to an accumulation of DNA strand breaks upon replication, causing apoptosis during the S phase of the cell cycle	Ovarian, lung, colorectal and pediatric cancer	Topotecan, irinotecan, belotecan
Podophyllotoxin and analogues	*Podophyllum* spp. (rhizome, roots) Alcohol extraction	Blockage of cell division metaphase of mitosis	Lymphomas and testicular cancer trials	No rentable
Roscovitine	*Raphanus sativus* (Radish) Chloroform extraction	Inhibition of cyclin dependent kinases; reduction of cell cycle progression	Phase II clinical trials in Europe	Roscovitine, seliciclib

**Table 2:** Examples of clinical trials carried out with the selected phytochemical compounds.

Compound	Type of Cancer	Main Results	Refs.
Sulforaphane	Prostate	Reduction of prostate-specific antigen after prostatectomy/Lengthening of the on-treatment prostate-specific antigen doubling time.	[75,76]
	Breast	Improved efficacy of doxorubicin, without any cytotoxic effect.	[77]
Resveratrol	Colorectal	Induction of apoptosis in malignant cells in hepatic metastasis.	[78]
	Breast	Reduction of DNA methylation of RASSF-1α and prostaglandin E2 expression.	[79]
	Prostate	Reduction of cancer recurrence.	[80,81]
Curcumin	Pancreas	Improved efficacy of gemcitabine, without any cytotoxic effect.	[82]
	Breast	Improved efficacy of paclitaxel, without any cytotoxic effect.	[83]
Quercetin	Gastric	High dietary intake is inversely related to the risk of cancer development.	[84]
Gingerol	Colorectal	Reduction of the risk of cancer development.	[85]
	Solid tumor	Enhanced antioxidant status of patients receiving chemotherapy/Improvement of general quality of cancer patients receiving chemotherapy.	[86,87]
Kaempferol	Ovarian	Reduction of the risk of cancer development.	[88,89]
	Pancreatic		

**Table 3:** Anticancer compounds obtained from plants.

Plant	Compound	Structure	Results (IC_50_ µM)	Refs.
*Aloe vera*	Emodin	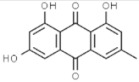	In vitro: Breast (8.6); Lung (19.6); Liver (12.8) Induction of apoptosis, specific energy-dependent pathway of drug incorporation.	[157,158]
*Artemisia annua*	Artemisinin	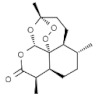	In vitro: Lung (0.9) Production of reactive oxygen species, inhibition of cell cycle in G0/G1 phase, induction of apoptosis, inhibition of angiogenesis.	[159]
	Artesunate	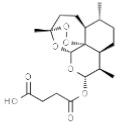	In vitro: Breast (2.3); colon (1.9); lung (9.8); leukemia (2); ovarian (15.0) Antiangiogenic effect; inhibition of VEGF expression.	[160,161,162]
*Betula* sp.	Betulinic acid	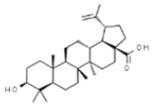	In vitro: Human leukemia (2.6); murine leukemia (2.1); cervix (2.5); colon (10.0); lung (4.3); skin (8.0); gastric (13.0) *In vivo:* (mice): Breast (13.5) Activation of extrinsic apoptosis pathway via up regulation of DR4, DR5 and PARP cleavage.	[163,164,165,166]
	Betulin	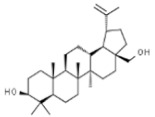	In vitro: Breast (30.7); colon (45.2); lung (45.2); leukemia (14.5); skin (6.8); prostate (17.9) Synergistic effect with chemotherapeutic drugs.	[167]
*Berberis vulgaris*	Berberine	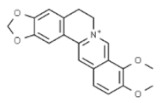	In vitro: Breast (25)/In vivo: Breast (25.0); colon (25.0) Induction of cell cycle arrest, synergistic effect with drugs/Reduction of tumor volume and angiogensis in a dose-dependent manner.	[168,169]
*Camellia sinensis*	Epicatechin gallate	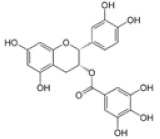	In vitro: Breast (350.0); colon (1000); lung (78.0) Induce apoptosis of cancer cell lines.	[170]
	Epicatechin	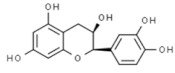	In vitro: Colon (60); prostate (8.9); ovarian (7.9) Growth inhibition.	[171]
	Epigallocatechin	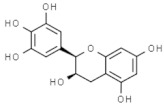	In vitro: Breast (22.0); colon (75.0); lung (70.0) Growth inhibition.	[172]
*Combretum caffrum*	Combretastatin	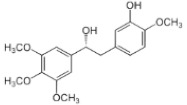	In vivo (mice): Breast (80-190); colon (8.4;) lung (7.1); leukemia (1.9) β-tubulin binding, causing the destabilization of the microtubules.	[161,173]
*Crocus sativus*	Crocetin	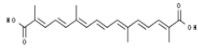	In vitro: Colon (0.2); lung (0.4); liver (0.6) Activation of Nrf2, reduction of lactate dehydrogenase A expression.	[174]
	Crocin	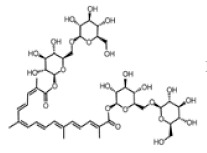	Inhibition of cancer cells’ proliferation or/and induction of apoptosis, both in vitro and in vivo studies.	[174,175,176]
	Safranal	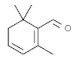	In vitro: Colon (20.0) Apoptosis and G2/M cell cycle arrest mediated by suppression of mTOR/PI3K/Akt pathway.	[177]
*Euphorbia peplus*	Ingenol mebutate	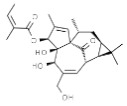	In vitro: Breast (23.9); colon (22.2); lung (15.2); skin (200.0); prostate (29.8) Necrotic mechanism.	[178,179]
*Gardenia jasminoides*	Geniposide	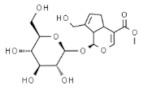	In vitro: Lung (351.5); medullobalstome (293.5) Activation of mitochondrial execution pathway by Caspase-9 and -3; increase levels of Bax in response to p38MAPK signaling initiation of the mitochondrial death cascade.	[161,180]
	Genipin	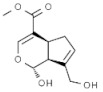	Inhibitor of mitochondrial uncoupling protein 2, induction caspase-dependent apoptosis in vitro and in in vivo models.	[181]
*Ginkgo biloba*	Ginkgetin	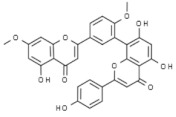	In vivo (mouse): Breast 10.0; colon 5.0; lung 10.0; prostate 5.0 p62/SQSTM1-mediated autolysosome formation and redox setting; inhibition of signal transducer and activator of transcription 3 activity.	[182,183,184]
	Bilobetin	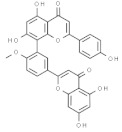	In vitro: Breast 57.6; lung 36.4; leukemia 46.0 Arresting the G2/M phase of the cell cycle.	[185]
	Isoginkgetin	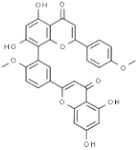	In vitro: Breast 91.2; lung 43.0; skin 18.8 Arresting the G2/M phase of the cell cycle.	[185]
*Glycyrrhiza glabra*	Licochalcone	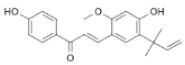	In vitro: Lung (30.0); skin (50.0); liver (44.13); gastric (42) Increased mRNA expression of DR3, DR5, caspases-3, caspases-8, caspases-10, Fas, Bad, Bax, Bcl-2, Bak, and PUMA; decreased level of PKCε, p70S6K, Akt.	[186,187]
	Licoagrochalcone	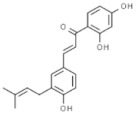	In vitro: Breast (28.6;) colon (25.4); leukemia (12.2); kidney (35.21) Induction of apoptosis, inhibition of proliferation.	[188]
*Matricaria chamomilla*	Apigenin	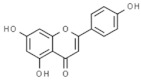	In vitro: Breast (100;) lung (93.7); liver (38.9) Up-regulation of DR5 pathway.	[189]
	Chamomillol	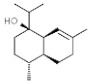	In vitro: Breast (300.0); colon (165.0); prostate (200.0); liver (300.0) Regulation angiogenesis by down regulating expression proteins.	[190,191]
*Panax ginseng*	Panaxadiol	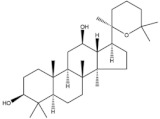	In vitro: Colon (25.0); liver (1.2) Regulation of cell cycle transition and the induction of apoptotic cells.	[192]
	Ginsenoside	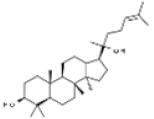	In vitro: Breast (30.0); colon (100.0); lung (50.0); skin (25–100) Induction of apoptosis, inhibition of proliferation.	[193]
*Papaver somniferum*	Noscapine	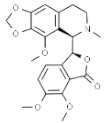	In vivo (mice): Breast (45.0); lung (73.0); leukemia (20.0); pancreas (70.0) Activating multiple signaling pathways including apoptosis.	[194]
*Persea americana*	Borbonol	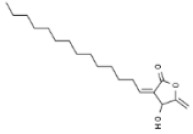	In vitro: Breast (20.5) Inhibition of proliferation.	[195]
*Salvia prionitis*	Salvicine	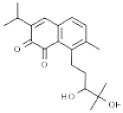	In vitro: Breast (1.4); leukemia (3.6); stomach (1.8) Breaks two strands of DNA by facilitating TOP2 activity; inhibits re-ligation	[196,197,198]
	Cryptotanshinone	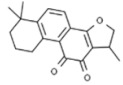	In vivo (mice): Breast (1.1;) lung (20.0); leukemia (4.8) Multispecific modes of actions: G2/M arrest, apoptosis, inhibition of cellular movement. Inhibition of NFκB orchestrates all these mechanisms.	[199,200,201]
	Salvianolic acid	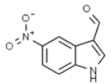	In vitro studies. Prompting apoptosis, disturbance of cell cycle and adjourning metastasis.	[202]
*Silybum marianum*	Silibinin	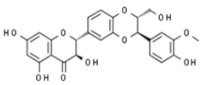	In vitro: Breast (24.0); skin (100.0); prostate (55.6); ovarian (25.0) Induction of apoptosis and cell cycle arrest.	[203,204,205]
	Silymarin	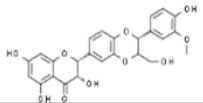	In vitro: Breast (75); prostate (67.9) Interference with expressions of cell cycle regulators and proteins involved in apoptosis; induction of cell cycle arrest.	[203,206,207]
*Zingiber officinale*	Paradol	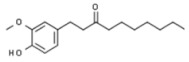	In vitro: Breast (20.4;) colon (10.4); leukemia (100); liver (>100). Reduction of cell viability.	[208,209]
	Shogaol	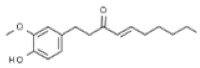	In vitro: Breast (24.4); colon (3.1); lung (25.8); liver (18.7) Inhibitory activity.	[209,210]
